# The effect of intra-nasal tetra sodium pyrophosphate on decreasing elevated nasal calcium and improving olfactory function post COVID-19: a randomized controlled trial

**DOI:** 10.1186/s13223-022-00711-0

**Published:** 2022-08-04

**Authors:** Mohamed H. Abdelazim, Ahmed H. Abdelazim, Waleed Moneir

**Affiliations:** 1 Department of Otolaryngology, Faculty of Medicine, Al-Azhar University, Damietta, 34518 Egypt; 2 Pharmaceutical Analytical Chemistry Department, Faculty of Pharmacy, Al-Azhar University, Nasr CityCairo, 11751 Egypt; 3 Department of Otorhinolaryngology, Faculty of Medicine, Mansoura University, Mansoura, 35511 Egypt

**Keywords:** Olfactory disorder, Calcium, COVID-19, Chelating agent, Tetra sodium pyrophosphate

## Abstract

**Background:**

Olfactory dysfunction is recognized as a symptom of COVID-19. Reports revealed the nasal calcium increase has adverse effects on olfactory function. Tetra sodium pyrophosphate, a chelating agent, can bind calcium and help improve olfaction.

**Methods:**

Sixty-four patients with olfactory dysfunction persisting for more than 90 days after COVID-19 were recruited. Participants were divided into 2 groups that received either 0.9% sodium chloride or 1% tetra sodium pyrophosphate for topical application. Olfactory function was tested with the Sniffin' Sticks test before treatment and 1 month later. In addition, nasal calcium was determined with an ion-selective electrode.

**Results:**

After topical application of tetra sodium pyrophosphate compared to sodium chloride, improvement from anosmia to hyposmia with decrease in calcium level was demonstrated. As for the results of tetra-sodium pyrophosphate, 81% showed improved olfactory function and 19% did not exhibit olfaction improvement.

**Conclusions:**

Intranasal application of tetra sodium pyrophosphate may be associated with improvement in olfactory function after COVID -19 infection.

*Trial registration* Ethical Committee of Damietta Faculty of Medicine approved this study on January 2021 (approval number, IRB 00012367-21-03-010).

## Background

The human olfactory process begins when volatile substances enter the nasal cavity and activate receptors in the olfactory epithelium. The olfactory receptor proteins are located in hair-like projections of olfactory neurons. Activation of the olfactory receptor proteins initiates a complex sequence of biochemical reactions [1]. Signals from olfactory receptor cells responding to volatile or odorant substances are picked up by the olfactory bulb [2]. The signal patterns leaving the olfactory bulb then travel to the piriform cortex. The output of the piriform cortex travels to various other brain regions and is finally interpreted in combination with other inputs as odor perception in the orbitofrontal cortex [3, 4].

The odorant triggered a calcium increase in the cilia of the neurons which affected excitation and adaptation [5]. The increase in calcium concentration in cilia was attributed to calcium entry through cyclic adenosine monophosphate gated channels. Calcium exerts an excitatory role by directly activating chloride channels. Activation of chloride channels by calcium leads to efflux of chloride from the cilia, which is accompanied by depolarization of the olfactory neuron [6]. Furthermore, calcium in conjugation with calmodulin mediates the process of odor adaptation by desensitizing cyclic adenosine monophosphate-gated channels. Restoration of calcium concentration to basal levels occurs via a sodium-calcium exchanger that expels calcium from the olfactory cilia [7]. Reports have demonstrated the increase of calcium in the nasal secretions of patients with olfactory disorders [8–10].

Olfactory dysfunction often occurs after a variety of upper respiratory tract infections. The pathophysiological mechanism of olfactory dysfunction in COVID-19 remains unclear. It has been hypothesized that a decrease in the sensitivity of sensory neurons and the co-expression of angiotensin converting enzyme 2 and transmembrane serine protease 2 in alveolar epithelial cells are the main causes of olfactory dysfunction [11]. On the other hand, some studies have suggested that conduction loss due to edema of the olfactory cleft, injury to the olfactory epithelium, and injury to the olfactory bulb itself are relevant causes of olfactory dysfunction [12].

Tetra sodium pyrophosphate (TSPP) is a chemical compound of pyrophosphate and sodium ions with known chelating properties. It is commonly used as a buffering and dispersing agent. TSPP is a safe chemical compound used in food additives and pharmaceutical products such as toothpaste. In addition, TSPP is a calcium chelating agent that has the ability to lower calcium concentration [13, 14]. It can chelate calcium from nasal secretions and form a stable complex product, which may be associated with an improvement in odor function.

Because studies of specific therapies for olfactory dysfunctions are sparse, the current study was designed to investigate the promising use of intranasal TSPP to improve olfactory disorders after COVID -19 through a prospective, randomized, blinded, controlled clinical trial. This is the first published clinical trial testing TSPP to improve olfactory dysfunction associated with COVID -19 infection.

## Materials and methods

### Study design

**Fig. 1:**
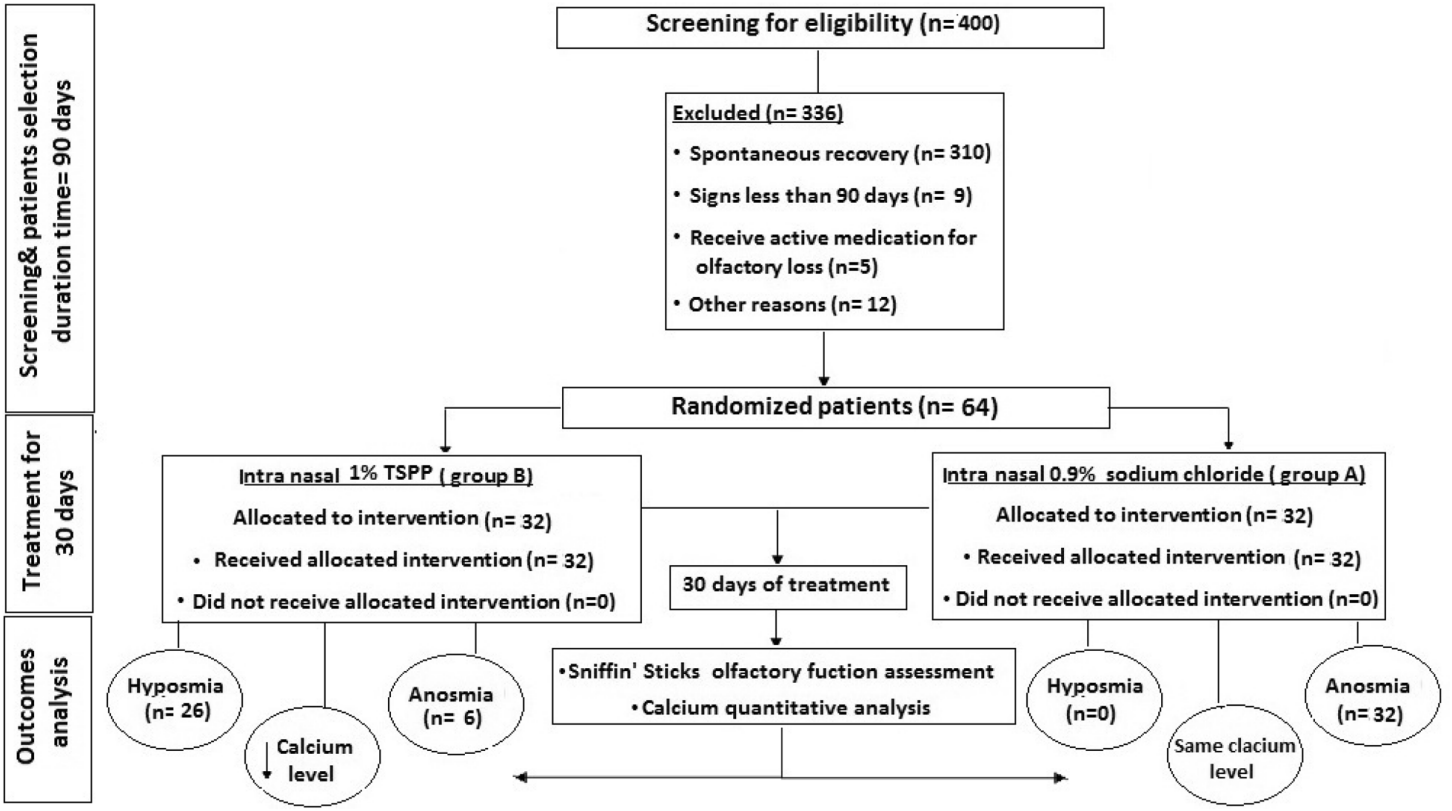
The flow chart of the described study

A prospective, randomized, blinded, double-controlled clinical trial was conducted from January 2021 to April 2021 in the Department of Otolaryngology, Damietta Faculty of Medicine, Al Azhar University, Egypt. The Ethics Committee of Damietta Faculty of Medicine approved this study (approval number: IRB 00012367-21-03-010). Prior to participation in the study, participants were informed by an assigned member of the study team about the aim of the study and the anticipated benefits and adverse effects. The participant signed informed consent statement form. After that, the patient received the blinded randomized proposed treatment. The flow chart of the proposed study is shown in Fig. 1.

### Sample size

In many reports, it was shown that 79.5% of patients recovered completely from the olfactory dysfunction through the first 2 months after COVID-19 infection [15]. Even considering the exclusion criteria, the sample rejection was up to 10%. A total of 400 patients were screened for eligibility. Thus, 64 patients were selected for the study and randomly allocated to 2 groups.

### Inclusion criteria

To be enrolled in this study the patients had to meet the following inclusion criteria: adults with previous COVID-19 infection confirmed by reverse transcription polymerase chain reaction (RT-PCR) in nasopharyngeal swabs, recovery from infection confirmed by at least two negative nasopharyngeal swabs, clinically confirmed signs of olfactory dysfunction persisted more than 90 days after SARS-CoV-2 negative testing. Only patients with measured olfactory augmented Sniffin' Sticks TDI test scores (threshold, discrimination and identification) ≤ 13, which represented only anosmia manifestations, were included in the study.

### Exclusion criteria

The exclusion criteria included: (1) patients with a history of previous olfactory dysfunction related to trauma or surgery; (2) patients with congenital olfactory loss and neurodegenerative diseases; (3) patients with psychiatric or neurological diseases; (4) patients who receive active medication for olfactory dysfunction; (5) patients with a history of adverse reactions to sodium salts; (6) pregnancy and (7) any patient with current participation in other COVID-19 trials.

### Study regimen procedures

#### Randomization

Patients were randomly divided to sodium chloride and TSPP groups by unratified block randomization with a block size of four. Computer generated randomization plan was developed to provide block randomization. Groups allocation and organization were blinded for the physicians and the patients. The record book remained in the hands of certain tem member who did not communicate with the patients or the interviewer.

#### Medication preparation

The Department of Pharmaceutical Analytical Chemistry, Cairo Faculty of Pharmacy, Al-Azhar University, Egypt presented the standard procedures for preparing the medications under the study. The medications were prepared as follows: group A, 0.9% sodium chloride intranasal spray; and group B, 1% TSPP in borate buffer solution with a pH of 8. As FDA has determined that TSPP is generally recognized as safe chemical compound and the commonly used concentration of TSPP may be up to 10% [16], so 1% TSPP was appropriate and safe to be used as topical nasal solution. The formulations were provided in identical opaque nasal spray bottles which deliver a standardized volume of 0.1 mL There was a specific sealed code for the assigned bottles and was not available to the team members involved in the study. This remained sealed, unless there was a need to recognize the secrecy due to any adverse effects presented by any of the patients.

### Treatment procedures

Patients were divided into 2 equal groups. The first group received intranasal spray 0.9% sodium chloride (group A). The second group received intranasal spray 1% TSPP in borate buffer solution with a pH of 8 (group B). Patients received their assigned bottle and were instructed to apply 2 sprays into each nostril three times daily for 1 month. An endoscopic examination of the olfactory cleft was performed. Improvement in olfactory function after intranasal treatment with sodium chloride and TSPP was clinically demonstrated. Patients were monitored for side effects throughout the study. Nasal secretions were also collected from all participants before treatment and one month later. Calcium concentration in nasal secretions was determined using a designed ion-selective electrode.

### Olfactory function assessment

The ‘Sniffin’ Sticks’ test (Burghardt^®^, Wedel, Germany) is a clinical evaluation of the olfactory function based on pen-like odor dispensers. It includes 3 tests of olfactory function, odor threshold (T), odor discrimination (D), and odor identification (I) [17, 18]. By measuring these tests, the TDI value could be determined. It was done before treatment and 1 month later. The patient was asked to identify an odor from a set of 4 visual and written cues. A TDI score of 30.75 points or more represents normosmia, a score between 16.75 and 30.50 points represents hyposmia and a score less than 16.75 points represents anosmia [19, 20].

### Determination of calcium in the nasal secretions

The nasal secretions were collected before treatment and 1 month later immediately after sneezing. The collection was done using a small stainless steel clamp (approximately 10 mm × 5 mm × 2 mm) on the septum between the nostrils to allow the secretions to drain into a special 1.5-mL tube [22]. The secretions were diluted by adding 2 mL borate buffer solution. The protein contents were denatured by adding 3 mL acetonitrile. Then, the solutions were evaporated to dryness and the residues were diluted with borate buffer solution in 10-mL volumetric flasks. Ion selective electrode using carbon paste [22, 23] was designed by the Department of Pharmaceutical Analytical Chemistry, Cairo Faculty of Pharmacy, Al-Azhar University. The designed electrode was used for the determination of the calcium concentration of the nasal secretions before treatment and 1 month later.

### Statistical analysis

The statistical assessment was done using SPSS v23 statistical software (SPSS, Inc, Chicago, Illinois). The results obtained were checked for normality and parametric or nonparametric tests were used accordingly. In general, results were expressed as mean ± standard deviation, unless otherwise stated. Differences in frequencies of the sample populations were assessed using Fisher’s exact probability test. Unpaired *t*-test, was used to compare and test the significance of the results of the 2 groups. Statistical significance was assigned when *p* < 0.05.

## Results

**Fig. 2:**
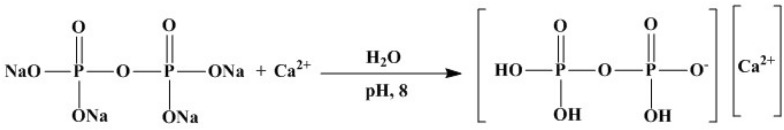
The chemical reaction pathway of TSPP and calcium

This prospective clinical trial introduced to test the application of TSPP as a topical nasal spray treatment for olfactory dysfunction post COVID-19 infection. The study included 64 patients with previously laboratory confirmed diagnosis of COVID-19 and clinically confirmed signs of olfactory dysfunction persisted more than 90 days after negative testing for COVID-19. The study group included 32 patients (18 females and 14males) with a mean age of 36.87 ± 5.25, and the control group included 32 patients (20 females and 12 males) with a mean age of 37.98 ± 6.27. The complete characteristics of the study participants were described in Table 1. Fisher’s exact probability test was used to assess the sample size. No significant difference was found between sodium chloride group and TSPP group, as shown in Table 1. TSPP had the ability to chelate calcium from the nasal secretions and mask the adverse effect of calcium on the olfactory function. The schematic reaction pathway between TSPP and calcium was shown in Fig. 2.

**Table 1:** Patient’s characteristics

Character	Sodium chloride	TSPP	*p *(Fisher exact probability test)
Sample size, n	32	32	–
Age (years), mean ± SD	37.98 ± 6.27	36.87 ± 5.25	–
Days since symptoms to enrollment, mean ± SD	96.54 ± 4.12	94.81 ± 3.89	–
Gender			
Male, n	14	12	0.79
Female, n	18	20	0.79
Smokers (current/never), n	5/32	4/32	1.00
Comorbidities, n			
Asthma	4	5	1.00
Diabetes	2	4	0.67
Hypertension	3	3	1.00
Migraine	4	5	1.00
Hyperuricemia	2	3	1.00
Current medication			
Anti-histamine	4	5	1.00
Metformin	1	2	1.00
Glimepiride	1	2	1.00
ACE inhibitor	3	3	1.00
Paracetamol	4	5	1.00

Olfactory function was clinically assessed using Sniffin' Sticks test before and after receiving sodium chloride and TSPP. Full monitoring of T, D, I also the TDI values were done. Mean T, D, I and TDI values before treatment and 1 month later were shown in Table 2. The results showed that the mean ± SD of the TDI value before and receiving sodium chloride was 11.10 ± 0.99 and 11.26 ± 0.72, respectively, Table 2. While, the mean ± SD of the TDI value before and after receiving TSPP was 11.28 ± 0.79 and 16.83 ± 2.13, respectively. In general, TDI values were compared to the reported reference values. Regarding to the measured olfactory scores of TSPP group, 26 patients (81%) showed an improved olfactory function from anosmia to hyposmia stage and 5 patients (19%) did not show improvement, Table 2. It could be due to the variation of the delivery efficiency for topical nasal sprays which expected that some patients with little to no response even to a highly efficacious drug. The results revealed that females scored higher than males, Table 2. Variation in medication adherence and administration technique could be contributing factors for these results. The change of TDI score (∆TDI) following treatment with sodium chloride and TSPP was calculated as following: (TDI score post treatment—TDI score pretreatment). The obtained results of the 2 groups was considered for the statistical comparison.

**Table 2:** Clinically recorded results with statistical assessment before and after treatment with topical sodium chloride and TSPP

	Sodium chloride			TSPP		
	Population size clinical assessment results					
	Pre administration	Post administration	*p*	Pre administration	Post administration	*p*
Population size with anosmia, n	32	32	1	32	6	**0.024**
Population size with hyposmia, n	0	0	1	0	26	**0.024**
Male population size with anosmia, n	14	14	1	12	4	**0.001**
Male population size with hyposmia, n	0	0	1	0	8	**0.001**
Female population size with anosmia, n	18	18	1	20	2	**0.0001**
Female population size with hyposmia,(n,%)	0	0	1	0	18	**0.0001**

	Measured Sniffin' Sticks TDI olfactory scores and measured calcium levels					
	Pre administration	Post administration	*t *(2.03)	Pre administration	Post administration	*t *(2.03)
T score, mean ± SD	2.34 ± 0.48	2.43 ± 0.50	1.79	2.41 ± 0.49	3.55 ± 0.64	**7.97**
D score, mean ± SD	4.47 ± 0.42	4.45 ± 0.41	1.69	4.48 ± 0.43	6.50 ± 1.13	**8.87**
I score, mean ± SD	4.29 ± 0.44	4.38 ± 0.45	1.69	4.39 ± 0.44	6.78 ± 1.16	**10.84**
TDI score, mean ± SD	11.10 ± 0.99	11.26 ± 0.72	2.01	11.28 ± 0.79	16.83 ± 2.13	**11.88**
Nasal calcium concentration (mM), mean ± SD	37.68 ± 1.80	36.40 ± 1.46	1.69	37.63 ± 2.14	29.18 ± 4.65	**10.13**
Mean ∆TDI ± SD	0.23 ± 0.36			5.41 ± 2.52		**11.25**
Mean ∆ calcium concentration ± SD	1.28 ± 1.02			8.44 ± 4.71		**8.39**

The significant difference indicated by the bold values, values in the parenthesis are the theoretical *t* values

Calcium concentration in the nasal secretions was quantitatively analyzed using carbon paste ion-selective electrode. Potentiometric determination of standard calcium samples with concentration range of 100–0.001 mM was done to obtain a standard calibration plot relating the electromotive force values to calcium concentration values. The designed electrode showed a Nernst slope of 29.12 mV/decade with a detection limit of 0.0001 mM. The calcium concentration of the nasal secretions was successfully determined using the designed electrode. The mean value of calcium concentration before treatment and 1 month later were shown in Table 2. The results showed that the mean ± SD of the measured calcium concentration (mM) before and receiving sodium chloride was 37.68 ± 1.80 and 36.40 ± 1.46, respectively, Table 2. While, the mean ± SD of the measured calcium concentration (mM) before and after receiving TSPP was 37.63 ± 2.14 and 29.18 ± 4.65, respectively. The results revealed remarked calcium level decrease in the patients received TSPP. The change of the measured nasal calcium concentration (∆ calcium concentration) following treatment with sodium chloride and TSPP was calculated as following: and the obtained results of the 2 groups was considered for the statistical comparison.

Unpaired *t* test is a statistical tool assessing whether there is a difference between two unrelated groups. It is used to compare the means of two samples when each individual in one sample is independent of every individual in the other sample. So, to test whether using intra nasal TSPP resulted in statistical significance improvement of the olfactory function in comparison to sodium chloride group, the change in the olfactory scores (∆TDI) of the patients treated with TSPP was compared to the same calculated values of sodium chloride group using unpaired *t* test. The result was statistically significant, *t* (32) = 11.25, *p* = 0.0008. Furthermore, the change of the measured nasal calcium concentration (∆ calcium concentration) of the patients treated with TSPP was compared to the same calculated values of sodium chloride group and the obtained results indicated the statistical significance, *t* (32) = 8.39, *p* = 0.0006. Full results were shown in Table 2. It could be concluded that the results of patients receiving intranasal TSPP showed a statistically significant difference with a relevant clinical improvement in olfactory function from the stage of anosmia to the stage of hyposmia. TSPP was generally well tolerated. Nasal discharge was the commonest side effect seen. However, mild burning sensation in either the nose or throat were also reported.

## Discussion

COVID-19 is associated with olfactory dysfunction in many patients. Several studies investigated the relationship between olfactory dysfunction after COVID-19 and the rate of recovery from loss of smell. It was reported that 79.5% of patients could expect complete recovery of olfactory function in the first 2 months. Also, 20.5% of patients could not achieve a normal level of olfactory function [15].

Although ions make up only 1% of nasal secretions [24], the ionic microenvironment in the olfactory cleft plays an essential role in the chemical-electrical transduction pathway to transmit olfactory information from the nasal lumen to the central processing system. During the response to odorants, there is a simultaneous increase in cyclic adenosine monophosphate and calcium concentrations. The increase in cyclic adenosine monophosphate is due to activation of adenylate cyclase. Similarly, the increase in intracellular calcium is due to influx into the cyclic nucleotide channels. The reports confirm the major role of calcium in olfactory receptor neurons and odor transmission mechanism [6–9].

Previous studies have investigated the use of intranasal sodium citrate as a calcium binder to reduce free calcium and improve olfactory function. The first study included 57 patients with various causes of hyposmia, and the identification scores of participants with only post viral hyposmia improved significantly [8]. In addition, a prospective clinical trial tested sodium citrate in 49 patients with post infectious olfactory dysfunction. The effect size did not reach clinical significance, although a statistically significant improvement in TDI values was observed after treatment with intra-nasal sodium citrate [9]. In another study, the use of intranasal sodium citrate was tested over a 2-week period in 60 patients. Although there was a significant increase in TDI scores, the improvement in quantitative odor function was not significant [10].

The current prospective randomized controlled trial tested topical use of TSPP for the treatment of olfactory dysfunction post COVID-19 infection. TSPP is a chelating agent which can sequester various metals and form complex products. In aqueous solution, TSPP had the ability to form stable complexes and lower the levels of the nasal calcium [13, 14]. The chelation process is mainly affected by pH and the presence of other competing cations. At pH 8, TSPP selectively forms the corresponding complex even in the presence of sodium, potassium or magnesium cations. This chelating action causes decrease in the concentration of calcium in the nasal secretions of patients.

The ‘Sniffin’ Sticks’ test is a universal tool used assess the human olfactory function. This test is used worldwide due to the reliability, accuracy and reproducible results. It provides the feasibility to evaluate the olfactory function by monitoring the odor threshold, odor discrimination and odor identification [17–20]. The results of TSPP receiving group indicated clinical improvement in the olfactory function from anosmia to hyposmia, as evidenced by the increase in the measured 'Sniffin' Sticks' test results.

The ion selective electrode provides accurate method for determination of calcium in the nasal secretions. Carbon paste electrode offers the advantages of fast response time and ease of use in a small volume of nasal secretions [25]. In the current work, determination of calcium concentration in the nasal secretions was done. Using the developed electrode, it was possible to quantify the calcium concentration before and after treatment. The results obtained showed decrease of calcium concentration in the patients who received TSPP. This could be due to the chelation effect of TSPP for calcium with formation of the corresponding complex product.

It could be concluded that there may be an association between the decrease of calcium concentration in the nasal secretions by the TSPP and the increase in the measured olfactory score with a subsequent improvement in the clinical olfactory performance.

This study has several limitations. The major limitation is the small sample size, which predisposed to an underpowered analysis. Also the need for additional studies is recommended to confirm the association between the changes of the nasal calcium concentration and the olfactory function. Actually, it will be important to extend this study for more studied populations, record observation over than 1 month. Also the testing the use of TSPP for olfactory dysfunction due to various factors rather than COVID-19 infection should be provided.

Finally, the effect of intranasal tetra sodium pyrophosphate to decrease the elevated nasal calcium concentrations in patients with olfactory disorders post COVID-19 was described. After treatment with tetra sodium pyrophosphate compared to sodium chloride, significant improvement from anosmia to hyposmia with decrease in calcium concentration of the nasal secretions was obtained.

## Data Availability

The datasets used and/or analyzed during the current study are available from the corresponding author on reasonable request.

## Abbreviations

TSPP: Tetra sodium pyrophosphate
T: Threshold
D: Discrimination
I: Identification
